# Real-world evidence study on tolerance and growth in infants fed an infant formula with two human milk oligosaccharides vs mixed fed and exclusively breastfed infants

**DOI:** 10.1186/s40348-023-00162-6

**Published:** 2023-08-19

**Authors:** Frank Jochum, Martina Meyer-Krott, Tina Hübler, Maja Lorenz, Raffi Bedikian, Joseph Zakarian, Anja Litzka, Guido Judex, Holger Hertzberg, Daniela Klee, Lothar Maurer, Martin Schacht, Adnan Al-Radhi, Jan Maier, Alexander Kröckel, Christian Faustmann, Luca Lavalle, Samir Dahbane

**Affiliations:** 1 Klinik für Kinder- und Jugendmedizin, Ev. Waldkrankenhaus Spandau Stadtrandstr. 555, 13589, Berlin und Medizinische Hochschule Brandenburg - Theodor Fontane (MHB), 16816, Neuruppin, Germany; 2 Kinderarztpraxis, Moses-Stern. Str. 28, 41236 Mönchengladbach, Germany; 3 Gemeinschaftspraxis Kinder- und Jugendarztpraxis, Clemensstraße 4, 47608 Geldern, Germany; 4 Kinder- und Jugendarzt, Venloer Straße 67, 41751 Viersen, Germany; 5 Kinder- und Jugendärztliche Gemeinschaftspraxis, Eugen-Zur-Nieden-Ring 1, 46145 Oberhausen, Germany; 6 Kinderarztpraxis, Suitbertusstr. 31, 40223 Düsseldorf, Germany; 7 Facharztpraxis für Kinder- und Jugendmedizin, Regensburger Str. 40, 93133 Burglengenfeld, Germany; 8 Zentrum für Kinder- und Jugendgesundheit Regensburg, Dr.-Leo-Ritter-Str. 4, 93049 Regensburg, Germany; 9 Kinder- und Jugendarztpraxis, Ludwigstraße 4, 91126 Schwabach, Germany; 10 Kinder- und Jugendarzt, Röntgen-Str. 6, 68642 Bürstadt, Germany; 11 Fachärzte für Säuglings-, Kinder- und Jugendmedizin, Welschgasse 39, 67227 Frankenthal, Germany; 12 Facharzt für Säuglings-, Kinder- und Jugendmedizin, Schwachhauser Heerstr. 63a, 28211 Bremen, Germany; 13 Kinder- und Jugendarzt Al-Radhi, Winckelhoferstrasse 3, 89584 Ehingen, Germany; 14 Kinder und Jugendarztpraxis, Geranienstr. 11, 70771 Leinfelden-Echterdingen, Germany; 15 Kinder- und Jugendarztpraxis, Schwarzwurzelstraße 52/54, 12689 Berlin, Germany; 16 Facharzt für Kinder- und Jugendheilkunde, Wiener Strasse 8a, 7400 Oberwart, Austria; 17 Nestlé Research, Société des Produits Nestlé S.A., Lausanne, Switzerland; 18 Global Medical Affairs, Société des Produits Nestlé S.A., Vevey, Switzerland

**Keywords:** 2′**-**Fucosyllactose, Lacto-N-neotetraose, Infant formula tolerance, Growth, Infant Gastrointestinal Symptom Questionnaire, Real-world evidence

## Abstract

**Introduction:**

Human milk oligosaccharides (HMOs) are important components of human milk having diverse functions in the development of infants. Randomized controlled trials (RCTs) have demonstrated that infant formulas with the HMOs 2′-fucosyllactose (2′FL) and lacto-N-neotetraose (LNnT) are safe, well-tolerated, and support normal growth. This study aimed to generate real-world evidence (RWE) on growth and gastrointestinal (GI) tolerance in infants consuming a formula with 1 g/L 2′FL and 0.5 g/L LNnT, including a mixed feeding group not studied before in RCTs.

**Participants and methods:**

This 8-week open-label prospective multicenter study was conducted in Germany and Austria, and included groups of healthy, exclusively breastfed infants (BF), exclusively formula-fed infants (FF) who received the HMO-formula, and infants mixed fed with both HMO formula and human milk (MF). Co-primary outcomes were anthropometry and gastrointestinal tolerance via validated Infant Gastrointestinal Symptom Questionnaire (IGSQ). Secondary outcomes included formula satisfaction and adverse events (AEs).

**Results:**

One-hundred six infants completed the study (46 FF, 22 MF, and 38 BF). Mean anthropometric z-scores were comparable between groups and generally within ± 0.5 of WHO medians at week 8. IGSQ composite scores demonstrated good GI tolerance in all groups with no significant group differences at week 4 or 8. IGSQ composite scores in FF improved during the course of the study and parents provided high satisfaction ratings for the HMO-formula. Four potentially product-related AEs were reported in FF (no in MF).

**Conclusions:**

In this RWE study examining an infant formula with HMOs, growth and GI tolerance outcomes were confirming the good tolerance and safety of this early feeding option previously reported in RCTs.

## Introduction

Human milk oligosaccharides (HMOs) are found in abundance in human milk and make up the largest solid component after lactose and lipids [1–4]. HMOs can become characterized as complex group of more than 200 different, nondigestible, and non-nutritional carbohydrates, providing an energy source for beneficial intestinal bacteria. There is evidence that HMOs improve the host defense by strengthening the gut barrier and immune-modulating effects and other mechanisms. Bovine milk, in contrast to human milk, contains relatively low levels of oligosaccharides, and the prevalence of fucosylated oligosaccharides, in particular, is quite low [5]. 2′-fucosyllactose (2′FL) is a trisaccharide composed of glucose, galactose, and fucose and is one of the most abundant HMOs. Levels of 2′FL vary depending on the secretor blood group status of an individual woman as well as ethnicity and stage of lactation, with 2′FL levels from about 0.9 to above 4 g/L in mature milk among secretors [6–14]. Another predominant HMO in human milk is lacto-N-neotetraose (LNnT) at levels ranging from 0.1 to 0.6 g/L with higher levels within the first month of lactation [7–10, 15–17].

Advancements in manufacturing technology now enable the synthesis of HMOs, and preclinical studies have established their safety for the purposes of supplementation of infant formulas [18, 19]. Safety, tolerance, and adequate growth as well as potential clinical benefits have been demonstrated in randomized controlled trials (RCTs) of term infant formulas supplemented with 2′FL alone and in combination with LNnT [20–22]. An RCT in the United States of America (USA) found that infants receiving formula supplemented with either galacto-oligosaccharides (GOS) or GOS + 2′FL demonstrated adequate growth and good tolerance [21]. Another RCT conducted in Belgium and Italy examined a study formula containing 1.0 g/L 2′FL and 0.5 g/L of LNnT in the test arms, while the control arm received standard formula without HMOs [20]. The HMO-supplemented formula was again well-tolerated and supported age-appropriate growth. A third study in the USA compared tolerance in infants receiving a 100% whey, partially hydrolyzed infant formula with the probiotic *Bifidobacterium lactis* with and without the further addition of 2’FL and found that the HMOs-supplemented formula was well-tolerated [22].

Evidence is emerging that HMOs play an important role in the development of a balanced intestinal microbiota and in supporting immune protection in breastfed infants [23–25]. Preclinical models have found that both 2’FL and LNnT promote the growth of *Bifidobacterium* species [26, 27]. Additionally, in a RCT of a term infant formula supplemented with 2’FL and LNnT, lower rates of parent-reported morbidity (particularly lower respiratory tract illnesses such as bronchitis) and lower use of antipyretics and antibiotics in the group receiving HMOs-supplemented formula were reported compared to the control infants [20]. Stool samples collected for microbiota assessment and metabolic signature at 3 months showed that the addition of 2’FL and LNnT shifted the stool microbiota closer to that observed in breastfed infants both in composition and function [28]. Collectively, these findings, in conjunction with the documented differences in HMOs composition between human and bovine milk, have provided a solid rationale for the benefits of bovine milk-based infant formulas with HMOs.

While the evidence provided to date in RCTs is supportive of the safety and tolerance of HMOs-supplemented infant formula, studies are needed in a real-world setting because results from a highly controlled RCT do not always translate outside of the trial setting [29]. Additionally, a relatively large proportion of infants in real-world settings are fed with both human milk and formula [30–32], a mixed feeding regimen not studied in RCTs. The current study was thus designed to complement and enhance existing RCTs by assessing the growth, safety, and tolerance of healthy term infants, consuming an infant formula supplemented with HMOs either exclusively or mixed with human milk in a real-world setting.

## Participants and methods

### Study design

This was a three group, non-randomized, open-label, prospective study in healthy, term (37–42 weeks of gestation) infants enrolled at age 7 days to 2 months. The study was conducted between 08/07/2019 and 24 July 2020, in 12 centers (pediatrician and adolescent doctors) throughout Germany and Austria. One study group included infants who were exclusively formula fed (FF), while a second group included infants who were mixed fed, i.e., received both formula and human milk (MF). The third group included exclusively breastfed infants (BF) serving as a reference population. Formula-fed infants were eligible to participate if their parent(s) had independently elected, before study enrollment, to formula feed. Breastfed infants were eligible if the infants had been exclusively breastfed since birth, and their parent(s) had decided to continue exclusively breastfeeding until at least 4 months of age. Exclusion criteria included any known intolerance/allergy to cow’s milk (formula-fed group only), conditions requiring infant feedings other than those specified in the protocol, and evidence of significant systemic disorders (cardiac, respiratory, endocrinological, hematologic, gastrointestinal, or other).

At study enrollment, FF and MF infants received the study formula and were fed for 8 weeks (56 days). Formula was prepared and fed at home and was given ad libitum. Infants completed an in-person clinic visit at enrollment (baseline) and again at day 56 ± 3 days (week 8 visit). A telephone visit with the parents was also conducted on day 28 ± 3 days (week 4 visit).

### Study product

Commercially available in Germany and Austria since autumn 2018, the study formula was provided to the participants at no charge. It was a partially hydrolyzed 100% whey, term infant formula with 67 kcal/100 mL consisting of 1.9-g protein, 11.5-g carbohydrates, and 5.1 g of lipids per 100 kcal powder, and with two HMOs: 1.0 g/L of 2′FL and 0.5 g/L of LNnT.

### Ethical approval and informed consent

This study protocol was approved by the ethics committee of the Berlin Chamber of Physicians. Prior to the conduct of any screening tests, informed consent was obtained from each participant’s parent. Good clinical practice was followed by all sites throughout the study. The study was registered with ClinicalTrials.gov NCT05150288.

### Study measures

At baseline and again at the clinic visit at week 8, anthropometry measures were obtained including weight, length, and head circumference using standardized procedures. Infant weight was measured without clothing or diaper on a calibrated electronic scale to the nearest 10 g. Recumbent length was measured on a pediatric length board to the nearest 1 mm. Head circumference was measured to the nearest 1 mm using a nonelastic plastic-coated measuring tape. Body mass index (BMI) was calculated as weight (kg)/(length (m))^2^. Z-scores for weight for age, length for age, head circumference for age, and BMI for age were calculated using the World Health Organization (WHO) Child Growth Standards [33].

The infant’s gastrointestinal (GI) symptom burden was assessed via the Infant Gastrointestinal Symptom Questionnaire (IGSQ) [34], a validated 13-item questionnaire that assesses GI-related signs and symptoms as observed by parents over the previous week in 5 domains: stooling, spitting up/vomiting, gassiness, crying, and fussing. Each item is scored on a scale of 1 to 5 with higher values indicating greater GI distress. A composite IGSQ score is derived from summing the individual scores with a possible range of 13 to 65 where higher values indicate greater GI distress and values ≤ 23 indicate no digestive distress [34]. The IGSQ was administered at baseline, week 4, and week 8.

A formula satisfaction questionnaire was administered to parents of infants in the formula-fed groups at week 4 and week 8 including three questions regarding the parent(s)’ experience with the study formula. Questions included the following: “Did your child like what he/she consumed?”, “How satisfied are you overall with the study product?”, and “Would you continue to provide the study formula to your child?”.

Adverse events (AE) were captured from the time of enrollment through the end of study. All AEs were assessed by the site investigator for duration, intensity, frequency, and relationship to study formula. AEs were classified by system, organ, and class (MedDRA SOC codes). In relation to published data of other studies, we expected AEs like the following: atopic diseases (f.e., eczema or cow’s milk protein allergy) infectious diseases; gastrointestinal symptoms, use of medication, and others.

### Statistical methods

Demographics and other baseline characteristics were compared between all pairwise combinations of feeding groups using two-sided Wilcoxon rank-sum tests for continuous variables and Fisher’s exact tests for categorical variables. Fisher’s exact tests were computed from contingency tables. For tables larger than 2 × 2, a Monte Carlo estimation of the exact *p*-value was performed with 20,000 samples; otherwise, a direct exact *p*-value computation was performed. Missing values were excluded before performing the aforementioned tests.

The co-primary outcomes were growth and composite IGSQ score. Feeding group comparisons were assessed individually at each time point (baseline visit and week 8 visit) for all growth measures using the analysis of covariance (ANCOVA) controlling for baseline value, age, gender, and study center. Listwise deletion was performed to handle missing values in the models. Tolerance was assessed via the IGSQ scores. The 13 individual questions in the IGSQ as well as the five domain scores and the composite IGSQ score were tabulated for each feeding group at each time point (baseline visit, week 4 visit, and week 8 visit). These scores were compared between the feeding groups individually at each time point using ANCOVA controlling for the baseline scores and age at baseline. The derived inferential statistics on the IGSQ scores were based on the sandwich estimator of the variance–covariance matrix of the models’ parameters due to some heteroscedasticity observed in the models’ residuals. Listwise deletion was performed to handle missing values in the models.

All analyses were conducted using SAS BASE 9.4/SAS STAT 15.1 on the SAS Life Science Analytics Framework (SAS LSAF, SAS Institute Inc., Cary, NC, USA) version 5.2.2. Due to the descriptive nature of the trial, no adjustment for multiple testing was performed. The statistical significance was assessed using an *α*-level of 5%.

Being a real-world evidence (RWE) study, sample size was based on practical and logistical feasibility and on the experience of published RCTs investigating safety and tolerance of formula containing 2′FL/LNnt [20–22]. The analysis set was defined by excluding infants who did not comply with the protocol (e.g., switched from breastfed group to formula-fed group and vice versa), were lost to follow-up, experienced tolerance issues, withdrew without explanations, or did not provide data due to other reasons. For BF, infants who were not exclusively breastfed and received other formula than the study product were excluded from the analysis set. All analyses of growth, tolerance, and satisfaction in this paper were conducted in the analysis set. AEs were reported for all enrolled infants.

## Results

### Subject disposition and demographics

**Fig. 1:**
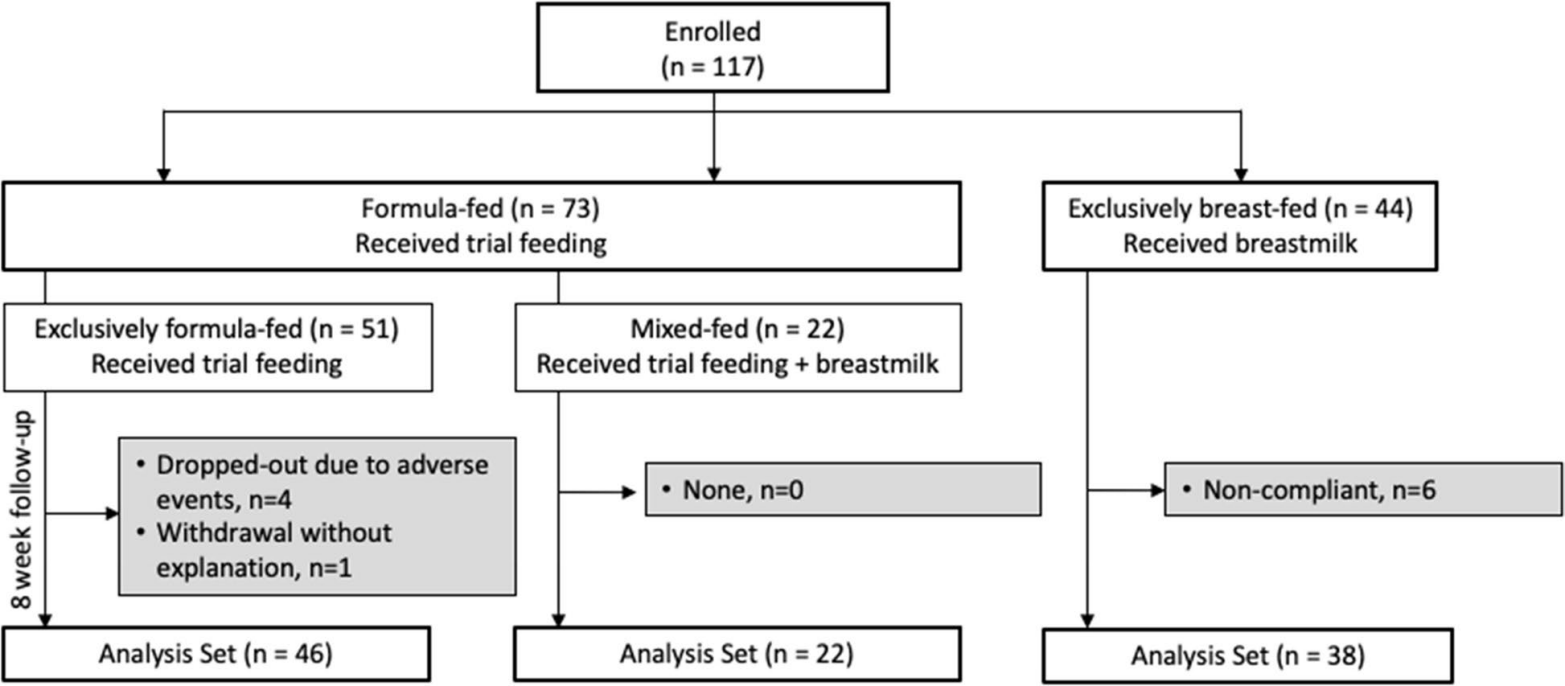
Flow chart of subject disposition

In this study, 117 infants were enrolled including 51 FF, 22 MF, and 44 BF infants (Fig. 1). The number of subjects in the analysis set was 46, 22, and 38, respectively, in FF, MF, and BF, with primary exclusion reasons being major protocol deviations (namely, breastfed infants who were fed a non-study formula at least once during the study) and intolerance issues.

The demographics and baseline characteristics of the infants included in the analysis set are shown in Table 1. MF was slightly younger at enrollment as compared with FF and BF. The gender distribution was comparable between groups. There were no differences between the three groups in terms of mothers’ ethnicity or educational attainment. Mothers of the infants in FF were the youngest. Fathers in FF had a slightly lower level of education than those in MF and BF. Parents in FF had significantly higher proportions of smoking compared to those in BF.

**Table 1:** Demographics and baseline characteristics by feeding groups in the analysis set (*N* = 106)Subject characteristics

	**BF**^**1**^ **(*N* = 38)**	**MF**^**1,2**^ **(*N* = 22)**	**FF**^**1,3,4**^ **(*N* = 46)**	***p*-value**^**1**^ **BF vs MF**	***p*-value**^**1**^ **BF vs FF**	***p*-value**^**1**^ **MF vs FF**
Age at enrollment, days (median [IQR])	43 (30 − 49)	33 (29 − 39)	39 (32 − 49)	0.097	0.706	0.067
Gender				1.000	1.000	1.000
Male	19 (50.0%)	11 (50.0%)	22 (47.8%)	-	-	-
Female	19 (50.0%)	11 (50.0%)	24 (52.2%)	-	-	-
Ethnicity				1.000	0.202	0.324
Caucasian	36 (94.7%)	21 (95.5%)	46 (100.0%)	-	-	-
Other	2 (5.3%)	1 (4.6%)	0 (0.0%)	-	-	-
Days breastfed since birth (median [IQR])	43 (30 − 49)	33 (29 − 39)	0 (0 − 5)	0.100	< 0.001	< 0.001
Days formula-fed since birth (median [IQR])	0 (0 − 0)	30 (16 − 32)	38 (30 − 49)	< 0.001	< 0.001	0.007
Mother’s age, days (median [IQR])	33 (30 − 36)	32 (29 − 35)	29 (26 − 34)	0.299	0.004	0.150
Mother’s highest level of education				0.600	0.202	0.194
Primary school	0 (0.0%)	0 (0.0%)	5 (10.9%)	-	-	-
High school	9 (23.7%)	5 (22.8%)	10 (21.7%)	-	-	-
College or above	18 (47.4%)	13 (59.1%)	17 (37.0%)	-	-	-
Professional school	11 (29.0%)	4 (18.2%)	14 (30.4%)	-	-	-
Mother smoked during pregnancy	1 (2.6%)	2 (9.1%)	10 (21.7%)	0.548	0.010	0.311
Father’s age, days (median [IQR])	34 (31 − 37)	36 (33 − 39)	31 (29–36)	0.160	0.040	0.006
Father’s highest level of education				0.664	0.012	0.043
Primary school	0 (0.0%)	0 (0.0%)	5 (10.9%)	-	-	-
High school	6 (15.8%)	4 (18.2%)	7 (15.2%)	-	-	-
College or above	18 (47.4%)	12 (54.6%)	10 (21.7%)	-	-	-
Professional school	14 (36.8%)	5 (22.8%)	17 (37.0%)	-	-	-
Missing, unknown, or less than primary school	0 (0.0%)	1 (4.6%)	7 (15.2%)	-	-	-
Father is a smoker	8 (21.1%)	9 (40.9%)	25 (54.4%)	0.132	0.002	0.430

Data shows as N and % unless otherwise noted. Percentages may not exactly add up to 100% due to rounding

*BF* exclusively breastfed, *MF* mixed fed, *FF* formula fed (exclusively), *IQR* interquartile range (Q1–Q3), *SD* standard deviation

^1^For continuous variables, *p*-values come from two-sided Wilcoxon rank-sum tests. For categorical variables, *p*-values correspond to Fisher’s exact tests

^2^Compared to BF, MF has been formula-fed longer (*p* < 0.001). There are no other significant differences between the two groups

^3^Compared to BF, FF has not been breastfed at all (*p* < 0.001) and formula-fed longer (*p* < 0.001). The mothers and the fathers in FF are younger (*p* = 0.004 and *p* = 0.040, respectively), and a greater number of mothers and fathers smoked more during pregnancy (*p* = 0.010 and *p* = 0.002). Fathers are also less educated in FF compared to MF (*p* = 0.012)

^4^Compared to MF, FF has not been breastfed at all (*p* < 0.001) and formula-fed longer (*p* = 0.007). The fathers in FF are younger (*p* = 0.006) and less educated (*p* = 0.043) compared to MF

### Growth

**Fig. 2:**
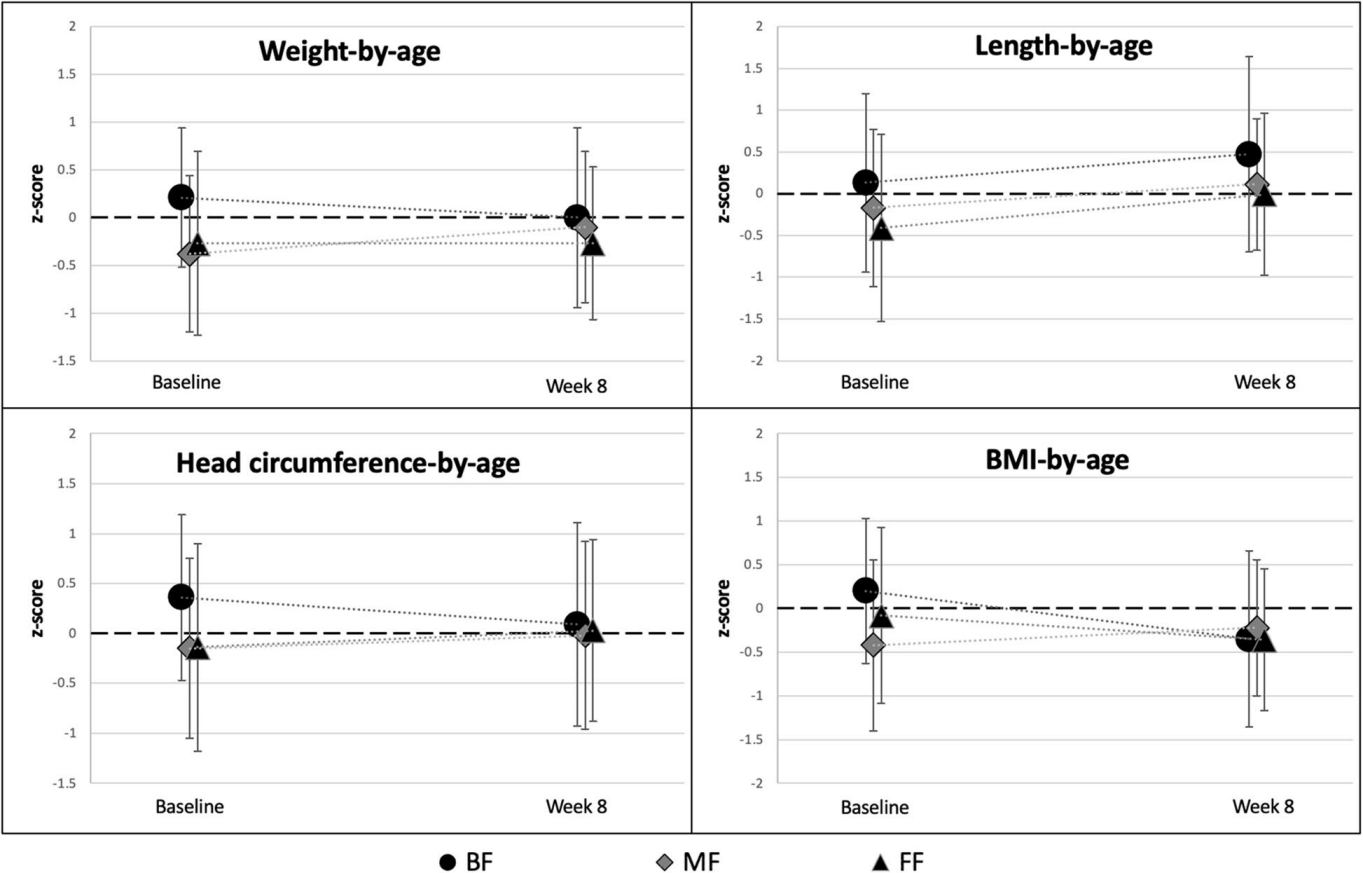
Anthropometric mean z-scores at baseline and week 8, by feeding group, analysis set (*N* = 106). Bars represent 95% confident intervals (two sided). BF, exclusively breastfed group; MF, mixed-fed group; FF, formula-fed group (exclusively)

Age-appropriate growth was observed in all three feeding groups. Baseline weight and length were slightly lower in MF. By week 8, there were no significant differences between any feeding groups for any of the anthropometric measures (all ANCOVA *p*-values between feeding groups > 0.05). Mean Z-scores for weight, length, head circumference, and BMI at baseline and week 8 are shown in Fig. 2. Weight-for-age, length-for-age, and BMI-for-age z-scores were comparable between all feeding groups at week 8. The mean z-scores were within ± 0.5 of the WHO medians at week 8. Head circumference-for-age z-scores were also comparable between groups and tracked closely with the WHO standards.

### Gastrointestinal tolerance

Table 2 shows descriptive characteristics for the five IGSQ domains and the overall composite IGSQ score. Composite IGSQ scores demonstrated low GI distress in all feeding groups at all time points. At baseline, FF had significantly greater GI distress compared to BF (mean difference [95% confidence interval (CI)] FF-BF = 5.18 [2.44, 7.91], *p* = 0.0003). However, there were no significant differences in the composite IGSQ score between any of the feeding groups at week 4 or week 8 suggesting that the GI tolerance in FF improved after introduction of study formula. For one of the five domains of the IGSQ (gassiness), there were no significant differences in scores between the groups at baseline, week 4, or week 8. There was a minor difference in fussiness between FF and BF at week 4 (mean difference [95% CI] = 0.821 [0.089, 1.554], *p* = 0.028), but no differences were observed at baseline or week 8. For the stooling domain, FF had significantly higher scores (i.e., more stooling issues) than BF at baseline (mean difference [95% CI] = 1.335 [0.543, 2.126], *p* = 0.001) but showed significant improvement by week 8 (mean difference [95% CI] =  − 0.151 [− 0.704, 0.403], *p* = 0.59), with FF moving closer to the stooling profile of BF. For the spitting-up/vomiting domain, FF again had significantly greater distress compared with BF at baseline (mean difference [95% CI] = 1.476 [0.457, 2.469], *p* = 0.005) and showed significant improvement at week 8 (mean difference [95% CI] =  − 0.128 [− 0.865, 0.609], *p* = 0.731), moving closer to the spitting-up/vomiting profile of BF. Lastly, FF had significantly greater distress for the crying domain compared with BF at baseline (mean difference [95% CI] = 0.951 [0.189, 1.713], *p* = 0.015) and week 4 (mean difference [95% CI] = 0.639 [0.047, 1.232], *p* = 0.035) and showed significant improvement at week 8 (mean difference [95% CI] =  − 0.339 [− 0.877, − 0.199], *p* = 0.214), becoming more comparable to BF.

**Table 2:** Composite IGSQ and domain scores^a^ at baseline, week 4, and week 8, by feeding group, analysis set (*N* = 106)

		**BF**				**MF**					**FF**				
		***N***	**Mean**	**SD**	**Min–Max**	***N***	**Mean**	**SD**	**Min–Max**	***p*-value**^**2**^	***N***	**Mean**	**SD**	**Min–Max**	***p*-value**^**2**^
**Composite IGSQ score**															
	**Baseline**	38	20.33	4.30	13–32	22	21.82	5.27	13–37	*0.279*	46	25.50	8.31	15–50	< *0.001*
	**Week 4**	38	19.20	4.05	13–29	22	20.45	4.73	13–32	*0.563*	43	21.94	5.64	13–37	*0.127*
	**Week 8**	38	18.76	3.97	14–32	22	18.14	3.67	13–25	*0.298*	45	19.08	5.49	13–36	*0.548*
**IGSQ domains**															
**Stooling**	**Baseline**	38	2.68	1.21	2–6	22	2.95	1.36	2–6	*0.769*	45	4.04	2.45	2–10	*0.001*
	**Week 4**	37	2.54	0.99	2–6	22	3.00	1.41	2–6	*0.364*	43	3.05	1.66	2–8	*0.549*
	**Week 8**	38	2.66	1.02	2–6	22	2.73	1.39	2–7	*0.994*	45	2.58	1.20	2–6	*0.590*
**Spitting up/vomiting**	**Baseline**	37	5.68	1.70	4–9	21	6.38	2.64	4–12	*0.293*	45	7.16	2.97	4–16	*0.005*
	**Week 4**	38	5.84	2.05	4–14	22	5.91	1.85	4–11	*0.758*	42	6.12	2.25	4–12	*0.949*
	**Week 8**	38	5.53	1.37	4–10	22	5.09	1.63	4–11	*0.183*	44	5.77	2.25	4–12	*0.731*
**Crying**	**Baseline**	38	4.16	1.22	3–7	22	4.32	1.46	3–8	*0.659*	46	5.11	2.28	3–13	*0.015*
	**Week 4**	38	4.00	0.96	3–6	22	4.09	1.48	3–8	*0.802*	43	4.91	1.99	3–10	*0.035*
	**Week 8**	38	4.37	1.20	3–8	22	3.95	1.33	3–7	*0.143*	45	4.24	1.65	3–11	*0.214*
**Fussiness**	**Baseline**	38	2.66	1.70	2–10	22	2.59	1.18	2–6	*0.931*	45	3.31	2.00	2–10	*0.095*
	**Week 4**	38	2.45	0.89	2–6	22	2.59	1.26	2–7	*0.522*	43	3.40	2.27	2–10	*0.028*
	**Week 8**	38	2.37	1.36	2–10	22	2.32	0.78	2–5	*0.928*	45	2.71	1.66	2–10	*0.420*
**Gassiness**	**Baseline**	38	5.11	1.75	2–9	22	5.45	2.18	2–9	*0.387*	45	5.82	2.06	2–10	*0.078*
	**Week 4**	38	4.37	1.55	2–7	22	4.86	1.58	2–8	*0.405*	43	4.44	1.76	2–8	*0.613*
	**Week 8**	38	3.84	1.75	2–8	22	4.05	1.65	2–8	*0.976*	45	3.80	1.80	2–7	*0.452*

*BF* exclusively breastfed group, *MF* mixed fed group, *FF* formula fed group (exclusively), *SD* standard deviation

^2^*p*-value compared to BF

^a^The IGSQ consists of 13 individual questions (grouped into five domains). Each question is scored on a scale of 1 to 5 with higher values indicating greater GI distress. The composite IGSQ score is derived by summing the individual scores, resulting in a possible range of 13 to 65 where higher values indicate greater GI distress. A composite *IGSQ* ≤ 23 indicates no digestive distress

For the individual items in the 13-item IGSQ, FF passed more hard stools than BF and MF and had more difficulties in passing bowel movements than BF at baseline. Still at baseline, FF compared to BF showed a higher number of times the baby arched its back in pain when spitting up, was crying during feeding, or it was not possible to stop the baby from crying and also a higher number of fussy days in the past week. In FF, total time spent crying and number of times unable to soothe baby’s fussiness were more than in MF. There were no significant differences between the groups for the other IGSQ individual items at baseline. At week 8, there were no significant differences in any of the individual items between BF, MF, and FF except the total time the baby spent crying. FF cried less than BF (mean difference [95% *CI*] =  − 0.303 [− 0.614, 0.007],* p* = 0.056) at week 8, but there were no other differences for the other crying items. Total crying time did significantly improve in FF between baseline and 8 weeks (mean difference [95% CI] =  − 0.444 [− 0.799, − 0.090], *p* = 0.015), while there was no change between baseline and 8 weeks for BF and MF.

### Formula satisfaction

Most parents in MF and FF reported that their child liked what he/she consumed, and that they would continue to provide the study formula to their child at both weeks 4 and 8 (Table 3).

**Table 3:** Formula satisfaction questionnaire results [N (%)], at weeks 4 and 8 among parents of infants receiving study formula, by feeding group, analysis set (*N* = 68 infants receiving formula)

	**MF**		**FF**	
	***N* = 22**	***N* = 22**	***N* = 43**	***N* = 46**
	**Week 4**	**Week 8**	**Week 4**	**Week 8**
How satisfied are you overall with the study formula?				
Satisfied	19 (86.4%)	17 (77.3%)	34 (79.1%)	39 (84.8%)
Neutral	2 (9.1%)	4 (18.2%)	7 (16.3%)	5 (10.9%)
Dissatisfied	0 (0.0%)	0 (0.0%)	2 (4.7%)	0 (0.0%)
Missing	1 (4.6%)	1 (4.6%)	0 (0.0%)	2 (4.4%)
Would you continue to provide the study formula to your child?				
Yes	21 (95.5%)	21 (95.5%)	37 (86.1%)	38 (82.6%)
No	0 (0.0%)	0 (0.0%)	6 (14.0%)	6 (13.0%)
Missing	1 (4.6%)	1 (4.6%)	0 (0.0%)	2 (4.4%)
Did your child like what he/she consumed?^a^				
Yes	21 (95.5%)	21 (95.5%)	39 (90.7%)	42 (91.3%)
No	0 (0.0%)	0 (0.0%)	4 (9.3%)	2 (4.4%)
Missing	1 (4.6%)	1 (4.6%)	0 (0.0%)	2 (4.4%)

Percentages may not exactly add up to 100% due to rounding

*MF* mixed-fed group, *FF* formula-fed group (exclusively)

^a^The question “Did your child like what he/she consumed?” was based on the parent’s observation when feeding their child (overall reaction, eagerness to consume the formula, refusal)

### Adverse events

A total of 46 subjects experienced 69 AEs during the course of the study, and no serious AEs were reported (Table 4). A total of 7.8% (*n* = 4) of the AEs were potentially formula related and were only reported in the FF Group.

**Table 4:** Overview of adverse events, by feeding group, in the safety data set (*n* = 117)

	**BF (*N* = 44)**	**MF (*N* = 22)**	**FF (*N* = 51)**
Occurrence	11 (25.0%) [18]	6 (27.3%) [10]	29 (56.9%) [41]
Severity			
Mild^b^	11 (25.0%) [18]	6 (27.3%) [9]	22 (43.1%) [32]
Moderate^c^	0 (0.0%) [0]	1 (4.6%) [1]	8 (15.7%) [9]
Caused study discontinuation			
Yes	0 (0.0%) [0]	0 (0.0%) [0]	4 (7.8%) [4]^a^
No	11 (25.0%) [18]	6 (27.3%) [10]	27 (52.3%) [37]

Data shown as *N* (%) [m]

*N* number of subjects with at least one AE, *m* total number of adverse events, *BF* exclusively breastfed group, *MF* mixed-fed group, *FF* formula-fed group (exclusively)

^a^These four infants in FF experienced potentially product-related AEs including instances of lactose intolerance, hard feces, vomiting, and diarrhea

^b^Mild AEs, negative clinical sign/situation which requires no intervention

^c^Moderate AEs, negative clinical sign/situation which requires an intervention but without relevant or long-lasting limitations for the subject

## Discussion

The results indicate that formula-fed infants, either exclusively or mixed fed, receiving the formula supplemented with 2′FL and LNnT, had age-appropriate growth in line with the WHO standards and comparable to BF infants. Growth was also comparable to that seen in previous studies with West and South European infant populations [35]. By week 8, GI tolerance as indicated by low IGSQ scores was comparable in the formula-fed infants with that in BF infants indicating the formula is well tolerated. The incidence of adverse events in all groups was low. As shown at Table 4, 7.8% (*n* = 4) of the AEs were potentially formula related. Despite the season of the year (fall-winter), cases of bronchitis were lower than expected from the literature [36]. Therefore, the results were not added as secondary outcomes.

The results of this RWE study are comparable to those from previous RCTs that have examined anthropometry and GI tolerance for term infant formulas supplemented with HMOs. One RCT was a multicenter, double-blind trial that enrolled 175 healthy term infants in Italy and Belgium at less than 14 days of age who were fed study formula for 6 months [20]. The HMO-supplemented formula (2′FL + LNnT) demonstrated age-appropriate growth as well as good tolerance as measured by parents. Another RCT included 189 term infants in the USA who were exclusively formula fed until 4 months of age [21]. Formulas with 2′FL (at two different dosages) and GOS were well-tolerated based on parental reports, and no significant differences were observed for growth compared to a control group. Notably, neither of those trials utilized a validated tool to assess tolerance, and thus, tolerance outcomes cannot easily be compared across studies. A recent RCT used the same validated IGSQ tool as in the current study to assess tolerance [22]. The HMO-supplemented formula was well tolerated as evidenced by similar IGSQ scores at week 6 between the groups with (mean [SD] = 20.9 [4.8]) and without (mean [SD] = 20.7 [4.3]) the addition of 2′FL. These scores are similar to those observed in FF in the current study at week 8 (mean [SD] = 19.1[4.5]). Additionally, a single-arm study of a formula supplemented with 2′FL fed to fussy infants showed significant improvement in IGSQ scores after 3 weeks of feeding (baseline mean [SD] = 34.1 [10.0]; week 3 mean [SD] = 21.4 [7.0]; *p* < 0.001) [37]. Although we did not limit our study to fussy infants, we also saw an improvement in IGSQ in FF in our study from baseline to week 8 (mean difference [95% confidence interval] =  − 6.639 [− 9.497, − 3.782], *p* < 0.0001). The improvement in GI tolerance in our study might be partially related to the natural evolvement of GI tolerance which improves with increasing age but could potentially also be attributed to the composition of the study formula including the two HMOs. Almost all infants switched to the study formula at the beginning of the study, i.e., they were receiving a different formula prior to enrollment (44 of 46 FF infants and 21 of 22 MF infants), suggesting that the HMO-containing study formula has better GI tolerance than the formulas without HMOs consumed prior to enrollment. Only one other real-world study has been conducted to our knowledge; a study with similar design to the current study was conducted in Spain and had very similar results for both growth and IGSQ scores [38]. The agreements between the previous RCTs, the Spanish real-world study, and the current real-world study are reassuring that growth, safety, and tolerance of HMO-supplemented formula are consistent and robust across different geographical populations.

This study has several strengths. First, GI burden was measured using a validated instrument, the 13-item IGSQ based on five separate domains of feeding tolerance. The use of a validated instrument provides information that is interpretable and meaningful to practicing clinicians. Second, this was an RWE study, a design distinct from the RCT, simpler, less restrictive, but still in line with current clinical practices, enhancing the generalizability of the results and providing complementary data to RCTs. The published prevalence of infants who are mixed fed indicates that at age 1 month, 30% of infants receive mixed feedings [30, 31], similar to that observed in this study. Thus, the demonstration of appropriate growth and good tolerance in the mixed feeding group of infants in this study provides important evidence not found in the RCTs conducted to date. Some limitations of the study should also be noted. An open-label, non-randomized design increases the risk for bias, in particular for response bias (even for validated questionnaires), and higher attrition rates and missing data. In a study with specifically defined feeding regimens such as ours, randomization is however not possible. The main aim of randomization is to have study groups with comparable characteristics. We therefore compared the baseline characteristics in our three groups, and there are no substantial differences except for infant age at enrollment and parents’ smoking status. Infant age at enrollment was slightly lower in MF compared with both BF and FF. As it may take longer for mothers to establish exclusive breastfeeding or formula-fed patterns, the younger age in MF at enrollment can be expected. To account for the slight difference in baseline age between the groups, we included baseline age in the statistical models to reduce potential risk of bias arising by the non-randomized nature of our trial. Smoking was higher among the parents in FF compared with BF which for the fathers could be linked to the slightly lower education level in FF compared with BF. Alternately, the parents in BF may have underreported their smoking habits to make a positive impression, a form of social-desirability bias. The study formula was supplemented with just a single level of 2′FL and LNnT, and thus, this study cannot assess whether the observed growth and tolerance effects might differ over a wider range of levels of these HMOs. Additionally, this study, while multicenter, took place within only two countries (Germany and Austria), and its results may not be generalizable outside of Northwest Europe. Furthermore, the authors want to point out that even if supplemented with HMOs, infant formula is not comparable with the gold standard “human breast milk” concerning multiple aspects. The aim of research in infant formula is of cause not to replace human breast milk — but to have the best possible substitute available in case breastfeeding fails or breast milk is not available.

In conclusion, this is one of the first studies to use real-world evidence to examine the supplementation of infant formula with HMOs. The results obtained were similar to those found in more tightly controlled RCTs, indicating robust effects for growth, safety, and tolerance in association with HMO-supplemented infant formulas.

## Data Availability

The data are available for research purposes, f.e., to perform meta-analyses.
